# How It Started...How It’s Going: From Decorating Carbon Nanotubes to Artificial Neurons, Beyond-Lithium Batteries, and Electrochemical Separations

**DOI:** 10.1021/acs.nanolett.5c04637

**Published:** 2025-10-10

**Authors:** Sarbajit Banerjee

**Affiliations:** † Laboratory for Battery Science, PSI Center for Energy and Environmental Sciences, Paul Scherrer Institute, Forschungsstrasse 111, CH-5232 Villigen PSI, Switzerland; ‡ Laboratory for Inorganic Chemistry, Department of Chemistry and Applied Biosciences, 27219 ETH Zurich, Vladimir-Prelog-Weg 2, CH-8093 Zürich, Switzerland

A shared journey in and from nanoscience intersecting time and again with the growth of *Nano Letters* as an interdisciplinary global forum for impactful science.

The turn of the century seemed like a time of giddy optimism...dot-coms were in full bloom, the mists of the Cold War had lifted, and buckyballs seemed poised to blur the distinctions between science fiction and reality. As an undergraduate at St. Stephen’s College in Delhi, I was inspired by the great sense of possibility that my instructors (many of them recent PhDs) conveyed about a nation on the move that stood ready to embrace the new millennium. Vibha Sharma, Rene Saksena, M. S. Frank, and Parvesh Sharma among others had a strong influence on my introduction to chemistry. We seemed to be on the brink of a new era where science and technology would bring “freedom and utterance”, as promised, to the aspirations of a billion people. In my college years, I had gained a great fascination for metals and was considering options for graduate school in coordination chemistry. The United States beckoned as a place of tremendous openness and excitement around science.

As a starting PhD student at Stony Brook University, I received an intriguing message from Stanislaus S. Wong who had just started as an Assistant Professor and was recruiting new students. He described some of his interests in scanning probe microscopy and the emerging discipline of nanoscience. This email led to many conversations that opened for me an entirely new world. I was the first PhD student in his research group. In the fall of 2000, we embarked on an ambitious agenda of exploring the fundamental reactivity and surface chemistry of carbon nanotubes with the idea of ultimately building more complex architectures.

The first year of graduate school was a blur. Between electron microscopes, synchrotrons, and lots of other fun toys, it seemed almost like we were laying the groundwork to a new discipline. In the summer of 2001, we started to put together a first couple of short letters describing metal coordination to carbon nanotubes and synthetic approaches toward building heterostructures of carbon nanotubes and quantum dots.^1^ *Nano Letters* had just launched and was already the intellectual home of this exciting new discipline of nanoscience and nanotechnology. The initial issues featured some of the most amazing work from my science heroes, so there was no question that this is where we would submit our manuscripts.

My first ever article carries a striking memory. We had printed the article on glossy paper in triplicate the night before and with some trepidation mailed it early in the morning on September 11th in a big envelope to Paul Alivisatos’s editorial office in Berkeley, California. In just a few hours, the world would change forever as the Twin Towers in Manhattan were attacked, and the world was plunged into uncertainty and despair. At some point in the week, we heard from ACS that they would prefer an online submission. We submitted the manuscript on the portal that would eventually become the Paragon Plus system on September 18th, 2001, and the first of the two articles appeared in print in October. Several months later, I remember riding the Long Island Rail Road into a much-changed New York City for a symposium on nanoscience organized by Charles Michael Drain at Hunter College where I had a chance to present a poster and to meet many of those that were leaders in this emerging new interdisciplinary area of science. I do not recall whether *Nano Letters* had formally sponsored the symposium, but there was a strong sense of the community having found a home in its pages. At Stony Brook and in the Stanislaus Wong group, I learned to think beyond conventional disciplinary boundaries and to bring diverse sets of tools and thinking to urgent problems, which has been a core philosophy for my research group. In particular, I picked up a great love for X-ray spectroscopy methods that have become a mainstay of my career.

During my postdoctoral training at Columbia University, I had the great privilege to approach nanoscience problems from a different, more physics-based perspective, under the tutelage of Irving P. Herman and to be a part of the Columbia Nanoscience center, which included Louis Brus, Horst Stormer, and many other former Bell Laboratories stalwarts. A lunch-time conversation (I cherish many excellent memories of such conversations) led to a whimsical and very fun article on mapping strain and fracture in CdSe nanocrystal films that also found a home in *Nano Letters*.^2^ This article and subsequent work with Irving and Jeffrey Kysar have inspired a lifelong interest in solid mechanics and strain engineering, which in recent times has been an active focus of my research group in the context of battery electrode and neuromorphic materials and led to sustained collaborations with the groups of Matt Pharr and Bai-Xiang Xu.^3^ Perhaps the most important lessons I learned at Columbia were to embrace scientific nomadism, a healthy irreverence for disciplinary boundaries, and to be unafraid of entering entirely new areas. This philosophy has been the inspiration for many forays of my own research group led by several intrepid graduate students into areas as diverse as space infrastructure, midstream transportation of fossil fuels, automotive coatings, and isotope separation, where we have had the opportunity to solve practical problems and create commercial value in parallel to our research programs in basic science.

I started my independent career at the University at Buffalo with a burning desire to understand phase transformations. I was part of a cluster of new hires in nanoscience and came into an institution where there was tremendous energy and, also, a strong commitment to societal impact. Collaborations with Sambandamurthy Ganapathy, David Watson, and Peihong Zhang have withstood the test of time and distance. A notable article stewarded by Christopher Patridge explored metal—insulator transitions in δ-K_*x*_V_2_O_5_ and was one of our foundational articles in what has become a mainstay of our research program in the design of *de novo* materials that emulate aspects of neuronal and synaptic function.^4^ Several of my first class of graduate students, Luisa Whittaker-Brooks, Jesus Velazquez, and Christopher Patridge, have gone on to illustrious careers that continue to touch upon nanoscience.

My own research journey has led me to directions oftentimes orthogonal to my initial training and along what appears to be a trajectory of increasing complexity in electrochemical materials spanning the spectrum from electrochemical energy storage to neuromorphic computing and critical materials recovery. In our current emphasis on growing large single crystals and deciphering their topochemical, electrochemical, and diffusionless transformations,^5^ I sometimes think I could not have come further from long hours spent in the laboratory working out nanocrystal syntheses. Yet time and again, there have been interesting intersections with *Nano Letters* and its interdisciplinary and ever-expanding community.

Looking back to when I started my own journey—leaving the comforts of home and family for graduate school several thousand miles away in a town where I knew no one at all—I was nervous, a little afraid, and awkward, but excited to explore all that the world of science had to offer. My journey has been much more than I could have ever imagined. It has taken me across continents, provided a glimpse of desperate need, given me a view of the tremendous promise that lies at the frontiers of technology, and imbued resilience in times of adversity. This journey has made me friends from all walks of life—whose own journeys continue to inspire and educate me. It has been an incredible ride in every sense. Even in our current time of unprecedented change and division, where perhaps some of the initial optimism has inevitably waned, science is the lens through which we make sense of the world around us, a vital enabler of our modern economies, and potentially the very tool that will lift vast populations out of poverty if access to discoveries can be appropriately democratized and disseminated. *Nano Letters* as a key part of the dissemination ecosystem will undoubtedly continue to play a vital role in this process.

Just in the last few months, my research group and I have started a new chapter at ETH Zürich and the Paul Scherrer Institut. We are excited for what the future holds and looking forward to forging new directions in electrochemical materials including next-generation batteries and sustainable resource utilization. Recent advances in our amazing constellation of experimental tools such as synchrotron light sources and neutron facilities hold promise to push up to and beyond current limits of energy, temporal and spatial resolution, and to enable unprecedented new insight into mechanisms of structure transformations we have long sought to explore. And so for *Nano Letters* and for us, in the words of President George H. W. Bush, “The new breeze blows, a page turns, the story unfolds, and so a chapter begins,”...one that I hope will bring to fruition the promise of science...not just for a few but for all.
